# Dataset of 37-subject EEG recordings using a low-cost mobile EEG headset during a semantic relatedness judgment task

**DOI:** 10.1016/j.dib.2025.111390

**Published:** 2025-02-12

**Authors:** Hannah Begue Hayes, Cyrille Louis Magne

**Affiliations:** Psychology Department, Middle Tennessee State University, Murfreesboro, TN, USA

**Keywords:** Mobile EEG, ERP, N400, Semantic processing, Psychometrics

## Abstract

This data article presents electroencephalography (EEG) data and behavioral responses from a study examining the efficacy of a consumer-grade EEG headset (InteraXon Muse 2) in measuring the N400 component, a neural marker of semantic processing. These data are linked to the article “Exploring the Utility of the Muse Headset for Capturing the N400: Dependability and Single-Trial Analysis”. Data were collected from 37 adult native speakers of English while they completed a semantic relatedness judgment task. Participants were presented with pairs of words and asked to judge whether the word pairs were semantically related (e.g., "pedal-bike") or unrelated (e.g., "icing-bike"). This dataset provides raw and preprocessed EEG data, alongside behavioral data (accuracy, response times) and comprehensive metadata. The MATLAB scripts for EEG analysis and the Python code for stimulus presentation and data acquisition are also included. These data offer a valuable resource for researchers interested in exploring the potential of consumer-grade EEG for language research. They can also be used to further investigate electrophysiological markers of semantic processing under different analysis parameters or in conjunction with other publicly available datasets.

Specifications Table

**Table:** 

Subject	Cognitive Neuroscience
Specific subject area	Mobile Electroencephalography, Psycholinguistics
Type of data	Behavioral and EEG data (.xdf, .set, and BIDS structure); experimental stimuli (.xlsx); custom-written Matlab code (.m, .mat); custom-written Python code (.py)
Data collection	EEG data were acquired using the InteraXon Muse 2 headset and streamed wirelessly via Bluetooth using the BlueMuse software and Lab Streaming Layer (LSL) protocol. Participants performed a semantic relatedness judgment task, with stimuli presented using PsychoPy software. Offline data preprocessing was conducted using EEGLAB and the Reliability Analysis Toolbox in MATLAB.
Data source location	Murfreesboro, Tennessee, United States, 35.8496° N, 86.3618° W
Data accessibility	Repository name: Open Science Framework (OSF) Data identification number: 10.17605/OSF.IO/U6Y9G Direct URL to data: https://osf.io/u6y9g/
Related research article	H.B. Hayes, C. Magne, Exploring the Utility of the Muse Headset for Capturing the N400: Dependability and Single-Trial Analysis, Sensors 24 (2024) 7961. 10.3390/s24247961

## 1 Value of the Data

• The data provide a unique assessment of a consumer-grade EEG headset's ability to capture the N400 component, a well-studied neural marker of semantic processing.
• This dataset can be reused to explore semantic-related ERP markers using diverse data processing and analysis approaches, enabling researchers to assess the influence of various parameters on the N400 effect identified in this study.
• The availability of raw EEG data allows for time-frequency analysis, facilitating the investigation of oscillatory brain activity linked to semantic processing, in addition to traditional ERP analyses.
• The dataset can be integrated with other publicly available datasets for conducting meta-analyses or for comparing the N400 across different experimental paradigms, populations, or EEG systems.
• All custom-written MATLAB and Python codes are provided to ensure the reproducibility of the data preprocessing and statistical analyses published in the original paper.

## 2 Background

This dataset was compiled to address a gap in the literature regarding the suitability of consumer-grade EEG headsets for language research, specifically for capturing the N400, an event-related potential (ERP) component commonly measured in language paradigms to study semantic processing [1]. The N400 is a negative-going deflection in the EEG signal that typically peaks around 400 milliseconds after someone encounters a word that is unexpected with the preceding context (e.g., "He spread the warm bread with socks"). By studying the N400, researchers can gain insights into various aspects of language comprehension and semantic expectation [1]. Furthermore, the N400 holds potential as a biomarker for various learning disabilities [2] as well as neurological and psychiatric conditions [3,4]. While previous research has validated the use of the InteraXon Muse 2 headset for measuring ERP components associated with basic sensory and cognitive processes [5,6], its efficacy in capturing smaller, language-related ERPs like the N400 remained unclear. This study represents a novel approach to investigating language processing by leveraging the affordability and accessibility of the Muse 2 EEG system. By demonstrating its effectiveness in capturing the N400 effect, we aimed to open new avenues for research in language and cognition, particularly in settings and populations traditionally underrepresented in neuroscience research.

## 3 Data Description

The data collected for the study presented in "Exploring the Utility of the Muse Headset for Capturing the N400: Dependability and Single-Trial Analysis" [7] is organized within an Open Science Framework (OSF) repository to ensure clarity and accessibility. The repository contains the following folders: Behavioral Data, EEG Data, Experimental Task, and Scripts.

Behavioral Data includes the output files from PsychoPy for each participant. Data are provided in both the native .psydat format and the more accessible .csv format. Each file contains the reaction time and accuracy rate for each stimulus collected during the semantic relatedness judgment task. This data can be linked to the corresponding EEG data using the subject ID code MUxx embedded in the file name, where xx denotes the participant ID number. The folder also includes a spreadsheet (Muse_Demograhics.xlsx) with demographic information for each participant (age, sex assigned at birth, race, ethnicity, handedness, and education level).

EEG Data includes two subfolders:

• Raw EEG: Individual participant EEG recordings in their original, unprocessed format are provided in the native open-source .xdf format from lab recorder, the more widely used .set format for EEGLAB, and the Brain Imaging Data Structure (BIDS) format. The xdf files provide a multi-modal record of the experiment, synchronizing the timing of stimuli presentation (via PsychoPy) with the participant's corresponding brain activity (via the Muse 2 EEG headset). Psychopy_stim_type Stream represents events generated by the PsychoPy software. The events in this stream indicate the timing and type of stimuli presented to the participant during the experiment. For example, the event Mismatch marks the onset of the prime word, MismatchTarget marks the onset of the target word, and Correct is the recording of participant responses. Muse-2B86 (00:55:da:b5:2b:86) Stream contains the EEG data collected from the 5 channels (4 EEG + 1 AUX) in the Muse 2 headband. Note that although the Muse 2 headset has 4 active EEG channels (typically corresponding to positions AF7, AF8, TP9, and TP10 based on the international 10-20 system), the data stream includes 5 channels. The first 4 channels are the EEG data. The fifth channel is from the AUX input, which is derived from a USB port connection. In this case, since the AUX channel was not connected to an external sensor, it only contains noise readings and should be disregarded during analysis. The .set file essentially mirrors the structure of the imported XDF data within EEGLAB. The core EEG structure houses the continuous 5-channel EEG data from the Muse headset, which is stored in the EEG.data field. Detailed information regarding each channel, including labels and location data, is contained within the EEG.chanlocs structure. Critically, events generated by PsychoPy, are stored in the EEG.event structure. The BIDS version of the dataset was generated from the .set files using the EEG-BIDS plugin (version 9.1) for EEGLAB, and subsequently validated for BIDS compliance with the online BIDS validator (https://bids-standard.github.io/bids-validator/). Within the BIDS structure, each participant's data subfolder (sub-muxx) contains the EEG data in .set format. Supporting files include a channel description file (sub-muxx_task-SemanticRelatednessJudgmentTask_channels.tsv) specifying channel labels and locations, and an event file (sub-muxx_task-SemanticRelatednessJudgmentTask_events.tsv) detailing stimulus timing and type. Metadata files (.json format) are also included to describe the experiment, acquisition parameters, and participant details.
• Processed EEG data is provided in two folders named Included and Excluded. The Included folder contains data from participants who met quality control criteria and were included in the final analyses. The Excluded folder contains data from participants who did not meet these criteria. All EEG data was preprocessed according to the methods described in Hayes and Magne [7] and the accompanying analysis scripts. This preprocessed data allows for immediate further analysis and exploration of the study's findings.

Experimental Task contains the complete set of word pairs used in the semantic-relatedness judgment task (.xlsx files) and the PsychoPy scripts to present the stimuli and record participant responses, as well as control EEG data acquisition from Lab Recorder and send event triggers with their associated timestamps to Lab Recorder (.psyexp files).

Scripts contains three subfolders with MATLAB scripts essential for EEG data processing and analysis.

• Preprocessing includes scripts for converting raw EEG data from .xdf format to EEGLAB's .set format, applying preprocessing steps to the raw EEG data (filtering, re-referencing, epoch extraction and artefact rejection), and creating an EEGLAB study to facilitate analysis of the data from all participants.
• The Stats folder contains scripts for conducting the robust statistical analysis of the EEG data reported in the manuscript. These scripts utilize the LIMO EEG plugin for EEGLAB. The scripts also implement a temporal clustering approach to control for multiple comparisons due to the high dimensionality of the data.
• Data quality provides MATLAB scripts used to assess the quality of the EEG data and determine whether a participant's data met the criteria for inclusion or exclusion in the final analyses.
  ○ A Script formats the preprocessed EEG data for compatibility with the ERA toolbox (ERP Reliability Analysis). This toolbox provides reliability estimates of ERP components, allowing for an evaluation of the data's internal consistency.
  ○ A script computes the root mean square (RMS) of the standard measurement error (SME) of the single-trial EEG epochs. This RMS value serves as a measure of the signal-to-noise ratio (SNR), providing an indication of data quality, as recommended for ERP analysis.

**Fig. 1:**
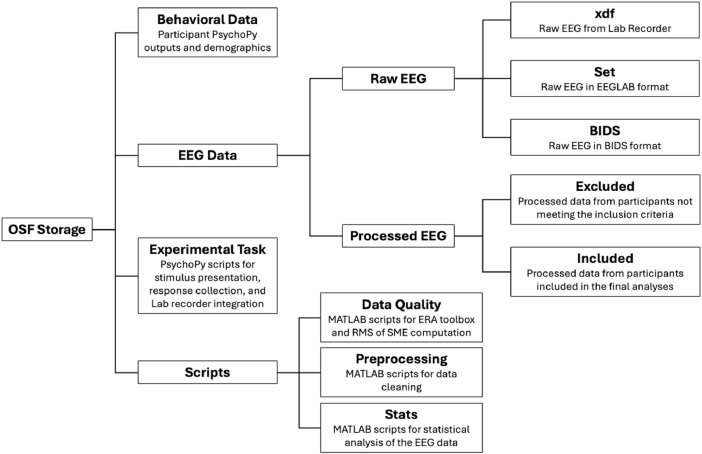
Nested Structure of folders and subfolders within the OSF repository (https://osf.io/u6y9g/).

Fig. 1 provides a visual representation of the organization of these files and folders within the OSF repository. This hierarchical structure ensures easy navigation and understanding of the data and its associated components.

## 4 Experimental Design, Materials and Methods

### 4.1 Participants

A total of thirty-seven first-year college students participated in the study for course credit. The mean age of participants was 19.95 years (SD = 3.81, range: 18-36), and the sample consisted of 51% females and 59% males (see Muse_Demograhics.xlsx in the Behavioral Data folder for each participant's demographic details). All participants had normal or corrected-to-normal vision and did not report any hearing deficits. The study received approval from the MTSU Institutional Review Board, and written informed consent was obtained from each participant.

### 4.2 Semantic-relatedness judgement task

Participants were presented with 112 pairs of words, comprising 56 semantically related pairs and 56 semantically unrelated pairs, displayed in a random order (see Experimental Task folder). The word pairs were adapted from a prior study [8]. The lexical frequency of each word was measured using log-transformed HAL frequencies provided by the English Lexicon Project database [9]. The log HAL frequency was 8.70 (SD = 1.94) for prime words and 10.50 (SD = 1.28) for target words. Additionally, the semantic relatedness of each pair was assessed using the Gensim python library and the pre-trained word embedding model "fasttext-wiki-news-subwords-300" to compute cosine similarity. The mean cosine similarity for related word pairs was 0.67 (SD = 0.11) and 0.30 (SD = 0.08) for unrelated pairs.

### 4.3 Experimental procedure

Participants were seated in a quiet room and fitted with a Muse headset, either self-placed or with researcher assistance. The headset position was adjusted to optimize the EEG signal quality, which was monitored in real time using a custom visualizer in MATLAB 2024a. Participants were instructed to judge the semantic relatedness of word pairs presented on a laptop positioned 3 feet in front of them. The experimenter concurrently monitored EEG data via an external monitor. Participants indicated their judgment by pressing "R" for related and "U" for unrelated on the laptop keyboard. They first completed eight practice trials followed by two blocks of 56 trials, separated by a short break. Each trial began with the presentation of the prime word for 500 ms, followed by a 500 ms blank screen, and then the target word, which remained until the participant responded. The task was implemented in PsychoPy [10], which handled the precise timing of stimulus presentation and recorded participant responses. Two sets of 112 word pairs were counterbalanced across participants to ensure all target words appeared in both related and unrelated word pair conditions while preventing word repetition within a set. Participants were randomly assigned to one of two counterbalanced lists. The experimental session lasted approximately 10 minutes, including headset setup.

### 4.4 EEG data acquisition and preprocessing

The Muse 2 headband includes two sensors behind the ears (TP9 and TP10), an electrode on each side of the forehead (AF7 and AF8), and a reference electrode in the center of the forehead (FPz). The open-source BlueMuse software [11] was used to connect the Muse headset to a Dell laptop via Bluetooth and to sample the raw EEG data at 250 Hz. The Lab Streaming Layer (LSL) protocol [12] was used to synchronize the timing of EEG data recording with event markers related to stimulus onset and participant responses, which were streamed by PsychoPy. Both the EEG data stream and the PsychoPy event stream were saved within a single time-aligned .xdf file using LabRecorder, the stream capture application included in the LSL ecosystem.

**Fig. 2:**
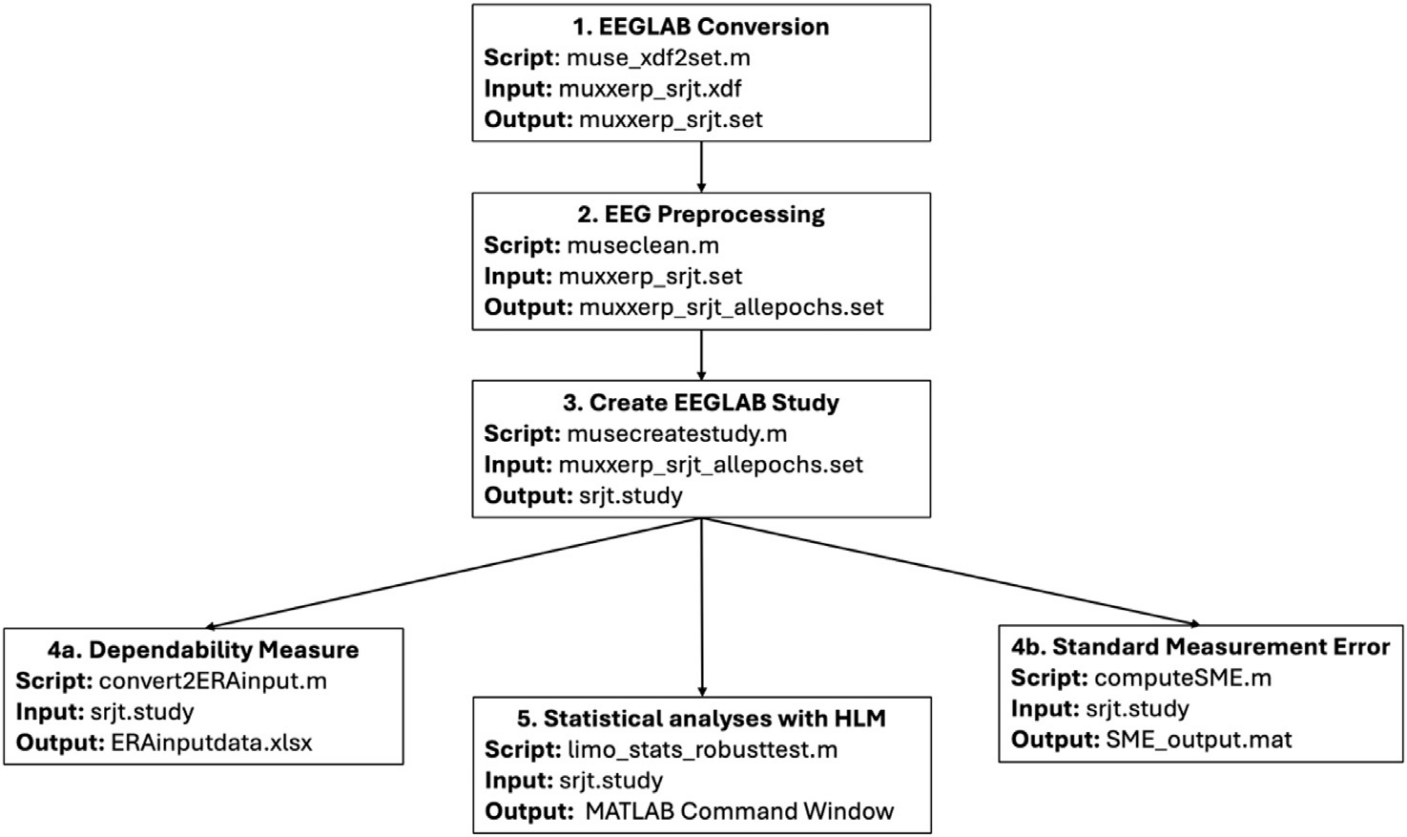
EEG processing pipeline from raw data to statistical analysis. For each step, the script used, the required input file type, and the generated output file type are provided.

Data processing was performed in MATLAB 2024a using the EEGLAB toolbox. The raw EEG signal was first high-pass filtered at 0.1 Hz and low-pass filtered at 30 Hz. Data were then re-referenced offline to the average of TP9 and TP10 due to their proximity to the mastoids, as N400 studies typically use an average mastoid reference [13]. Next, EEG epochs were extracted from -100 ms to 900 ms relative to the target word onset. Baseline correction was performed using the 100 ms to 0 ms pre-onset average. Epochs exceeding ±75 µV were rejected, and only trials with correct responses were included in the final analyses (see Fig. 2 for processing pipeline). The mean number of trials retained in the final dataset after preprocessing was 43.2 (SD = 9.0, range: 24-55) for the semantically related condition and 42.9 (SD = 8.3, range: 27-54) for the semantically unrelated condition.

### 4.5 Statistical analysis

**Behavioral Data.** Analyses were conducted in Jamovi version 2.5, a free and open-source statistical package. Accuracy rates were analyzed using a generalized linear mixed model (GLMM) with a binomial distribution and a logit link function. Response times (for correct responses only) were analyzed using a GLMM with a Gamma distribution and a log link function. The fixed effect was condition (semantically related and semantically unrelated), while participants were included as a random effect with a random intercept.

**N400 Reliability**. We used generalizability theory to calculate dependability, a measure of internal consistency analogous to Cronbach's alpha, to assess the internal consistency of the Muse EEG data within the N400 latency range. Dependability estimates were computed with the ERP Reliability Analysis (ERA) MATLAB Toolbox v0.4.5 [14] for each experimental condition within the 250–600 ms time window. Following established guidelines [15], we set a reliability threshold of 0.70 to determine the minimum number of trials required for a participant's data to be included in the statistical analysis.

To evaluate the quality of our ERP data, we adopted the approach recommended by Luck et al. [16] and utilized the root mean square (RMS) of the standardized measurement error (SME). The SME, calculated for each participant, represents the standard deviation of the single-trial mean amplitudes divided by the square root of the number of trials. By computing the RMS of these individual SME values across all participants, we obtained the RMS-SME, which reflects the variability of the N400 mean amplitude across the sample. A lower RMS-SME value thus indicates higher data quality. The RMS-SME was 0.96 for the semantically related condition and 1.00 for the semantically unrelated condition.

**Semantic Relatedness N400 Difference.** To compare the single-trial N400 responses in the semantically related and semantically unrelated conditions, we implemented hierarchical linear modeling with the LIMO EEG plug-in for EEGLAB [17]. First, we accounted for within-subject variability across single trials by using ordinary least squares (OLS) regressions to estimate the beta parameters for each condition at each time point and electrode. Next, Yuen's robust paired t-tests were computed at the group level to compare beta estimates between the two conditions, and a non-parametric temporal clustering approach was used to correct for multiple testing, controlling the family-wise error rate at an alpha level of 0.05 [18]. This approach involved generating null distributions through a permutation procedure with 1000 iterations [18,19] to obtain univariate p-values and cluster-forming thresholds. Finally, cluster-based inference was performed using a cluster-sum statistic based on squared t-values [17,18].

## Limitations

A notable limitation of this dataset is the use of an EEG headset equipped with only four electrodes. While this choice facilitates a more portable and user-friendly setup, it compromises the spatial resolution of the data compared to both research-grade EEG systems and certain consumer-grade devices that feature a higher number of electrodes. As a result, this may affect the accuracy of source localization and the ability to detect subtle or localized brain activity. Furthermore, a few participants displayed significant movement artifacts in their EEG recording, despite efforts to minimize head movement during the experiment. These recordings may contain noise that could potentially impact the analysis. Nevertheless, these datasets are made available in the Excluded subfolder of Processed EEG data. This ensures participant inclusivity and offers researchers the opportunity to examine artifact correction techniques or to utilize the portions of the recordings that remain unaffected.

## Ethics Statement

The research was carried out in accordance with The Code of Ethics of the World Medical Association (Declaration of Helsinki). The protocol was approved by the Institutional Review Board (IRB) of Middle Tennessee State University (protocol number: IRB-FY2023-173, 31 May 2023). All participants provided written informed consent.

## CRediT authorship contribution statement

**Hannah Begue Hayes:** Conceptualization, Methodology, Software, Validation, Data curation, Writing – original draft, Writing – review & editing. **Cyrille Louis Magne:** Conceptualization, Methodology, Software, Validation, Data curation, Visualization, Writing – original draft, Writing – review & editing, Supervision, Resources, Funding acquisition.

## Data Availability

Open Science FrameworkMuse Validation for Language ERPs (Original data)
